# Attraction of female house mice to male ultrasonic courtship vocalizations depends on their social experience and estrous stage

**DOI:** 10.1371/journal.pone.0285642

**Published:** 2023-10-10

**Authors:** Jakob Beck, Bettina Wernisch, Teresa Klaus, Dustin J. Penn, Sarah M. Zala

**Affiliations:** Department of Interdisciplinary Life Sciences, Konrad Lorenz Institute of Ethology, University of Veterinary Medicine Vienna, Vienna, Austria; Belgrade University Faculty of Medicine, SERBIA

## Abstract

Male house mice (*Mus musculus*) produce complex ultrasonic vocalizations (USVs), especially during courtship and mating. Playback experiments suggest that female attraction towards recordings of male USVs depends on their social experience, paternal exposure, and estrous stage. We conducted a playback experiment with wild-derived female house mice *(M*. *musculus musculus)* and compared their attraction to male USVs versus the same recording without USVs (background noise). We tested whether female attraction to USVs is influenced by the following factors: (1) social housing (two versus one female per cage); (2) neonatal paternal exposure (rearing females with versus without father); and (3) estrous stage. We found that females showed a significant attraction to male USVs but only when they were housed socially with another female. Individually housed females showed the opposite response. We found no evidence that pre-weaning exposure to a father influenced females’ preferences, whereas estrous stage influenced females’ attraction to male USVs: females not in estrus showed preferences towards male USVs, whereas estrous females did not. Finally, we found that individually housed females were more likely to be in sexually receptive estrous stages than those housed socially, and that attraction to male USVs was most pronounced amongst non-receptive females that were socially housed. Our findings indicate that the attraction of female mice to male USVs depends upon their social experience and estrous stage, though not paternal exposure. They contribute to the growing number of studies showing that social housing and estrous stage can influence the behavior of house mice and we show how such unreported variables can contribute to the replication crisis.

## Introduction

House mice (*Mus musculus)* emit ultrasonic vocalizations (USVs) during courtship and mating, and the number of USVs emitted during sexual interactions correlates with males’ mating and reproductive success [1,2]. USVs have been described in wild-derived mice [3,4] as well as laboratory mice [5–9] in a variety of contexts. They are emitted as discrete sounds ("calls") that can be automatically detected [10] and classified into several different classes (at least 3 to 12 "syllable" types), according to their shape, complexity, and other spectrographic features [11]. Male courtship USVs are uttered in repeated phrases that vary in syllable sequences ("syntax") and several other features comparable to the songs of birds and cetaceans [9]. Both sexes emit USVs during opposite-sex interactions [12,13] and their calls share similar qualitative features [13], though males produce most calls (ca. 85% to 93%) during opposite-sex interactions [13,14]. Male USVs are individually distinctive [15,16] and differ among laboratory strains [17]. Yet, males also modulate the emission of courtship USVs depending upon several factors, including their health [18], social housing [19], socio-sexual experience [20–22], sex of the stimulus individual [13], and estrous stage of a stimulus female [23]. Females’ responses towards male USVs have been investigated using playback experiments [2,4,24–29]. Females exhibit greater vocal responses to USVs than do males [26], and they are more attracted to complex than simple types of male USVs [25]. Females show more attraction to the USVs of males of their own species than towards an unfamiliar *Mus* species [27] and towards USVs of unfamiliar non-kin versus familiar male siblings [4]. The aim of our present study was to investigate factors proposed to influence female attraction to male USVs.

Three different factors have been suggested to influence female attraction to male USVs (which are similar to factors proposed to influence female attraction to male scent [30,31]): Firstly, habituation responses of female (CBA) mice to male USVs were influenced by whether they were kept in individual housing (IH) versus social housing (SH) [32]. Housing did not influence female attraction to USVs versus white noise, but IH mice and SH (with females) surprisingly showed increased attraction over time, whereas SH mice (with males and females) showed no increase over time. Social housing has been found to influence several behaviors in mice and other rodents [33], including sexual behaviors and auditory mechanisms that could alter female responses to USVs. For example, male mice [34] and rats (*Rattus norvegicus*) [35] kept in IH show lower sexual motivation than SH males, and female mice socially isolated during puberty exhibit less receptive sexual behaviors (lordosis) compared to socially reared individuals [36]. Mice kept in IH versus SH show differences in auditory perception [37], including perception of USVs [38], and neural auditory mechanisms [39–41]. These findings are interesting, but also concerning because studies on laboratory rodents rarely provide information about social housing conditions, and such unreported variables potentially contribute to the replication crisis [42–45].

Secondly, another playback study found that female attraction to USVs of males from a different strain depended on whether females had been reared with their father (paternal exposure or +PE) or not (–PE) (C57BL/6 and Balb/C mice) [28]. Females were preferentially attracted to the USVs of males from strains that differed from their foster father, whereas females reared without a father lacked preferences. These results support the hypothesis that females learn characteristic features of their father’s USVs (sexual imprinting), as a mechanism to avoid inbreeding [4]. This hypothesis predicts that neonatal paternal exposure will enhance female preferences for the USVs of unfamiliar males. These results also raise the possibility that neonatal exposure to the USVs of an adult male are necessary for females to recognize and develop a normal response to male USVs (of any male). Studies on the effects of early social experience on behavior are needed, especially since PE and other rearing conditions are rarely reported in studies on laboratory rodents.

Thirdly, this same playback study found that females only showed preferences for male USVs of other strains when they were in diestrus (when they are not sexually receptive) rather than in estrus (when females become sexually receptive) [28]. This result is surprising since estrous females are expected to show an enhanced rather than a cessation of attraction towards courtship signals. Yet, a previous study with wild house mice found estrous females were attracted to male USVs [4]. Estrous stage is another factor not usually reported in studies on laboratory rodents, and its effects on behavior are unclear and controversial [46–48].

We conducted playback experiments with wild-derived house mice (*Mus musculus musculus*) to examine the three factors proposed to influence female attraction to male USVs, which included: (1) social- versus individual-housing; (2) neonatal paternal exposure or not; and (3) proestrous and estrous (sexually receptive) stages versus metestrous and diestrous (unreceptive) stages. Females were simultaneously presented with a USV playback stimulus and a control (the same recording with USVs removed, leaving only the background noise). Trials were conducted in the absence of olfactory stimuli or direct male exposure, and level of females’ attraction to the two different acoustic stimuli was recorded. We expected that social housing (SH) and paternal exposure (+PE) would enhance female preferences for male USVs. Given that male USVs are courtship signals [3–9], we anticipated that females would show pronounced preferences during pro-estrus and estrus (when they are sexually receptive); however, it is also possible that they only show attraction to male USVs during diestrus (when they are not receptive) [28]. Finally, we recorded female habituation to male USV playbacks to determine whether the decreased interest in playbacks of male USVs over time found in most studies [4,26,28,29] depends on social housing [32].

We use the term “individual housing” rather than “social isolation,” because the mice were not socially isolated in the strict sense (as they were exposed to vocalizations and other sensory cues from mice in our colony). Also, we do not refer to social housing as the "control", as both types of housing are artificial treatments, and neither are controls. IH and SH are both artificial conditions that lie in between two ends of a continuum of social conditions studied in rodents, from complete sensory and social isolation in artificial laboratory conditions on one end (social isolation *sensu stricto*) to social housing with both sexes in seminatural conditions at the other end (e.g., see S1 Fig in [69]).

## Material and methods

### Animals

This study was approved by the Ethics and Animal welfare Committee of the University of Veterinary Medicine, Vienna in accordance with the University’s guidelines for Good Scientific Practice and authorized by the Austrian Federal Ministry of Education, Science and Research (BMBWF 2021–0.588.540) in accordance with current legislation. We conducted our study with 48 wild-derived female house mice (F2 from wild-trapped *Mus musculus musculus*) as test subjects (aged 202–332 d), which were all virgins. We used females from 20 different families (mean: 2.4, range: 1 to 7 females per family), no more than 2 females from the same litter, and we avoided using females from the same litter within each treatment (this was possible for 16/20 families). More than half (26/48) of the females had been used in one or two previous experiments (i.e., 6 females were used by placing them in an empty cage to collect voided urine, 11 were used by briefly presenting them to a male behind a divider to provide a sexual stimulus, and 9 participated in both studies). Three to six months passed between the females’ prior use and the present study, and we assume that these experiences had no effects on their attraction to USVs. We used recordings from 12 male mice (F2 generation), which had no previous sexual experience. All mice were housed in standard colony conditions on a 12/12-hour light/dark cycle (dark period begins at 15:00 h). Food and water were provided *ad libitum* with bi-weekly cage changes and individuals were earmarked for identification. All mice lived in standard mouse cages (mouse cage type IIL, 36.5 x 20 x 14 cm, Tecniplast, Germany). Each cage contained aspen bedding (ABEDD, Austria), nesting material (Nestlet, Ehret, Austria), a translucent red house (Tecniplast, Germany) and a cardboard tube. At every cage change, 5 g of seeds and 5 g of apple were added to the cage as additional enrichment.

### Social experience treatments (rearing and housing)

We manipulated both paternal exposure and social housing, and we consider both treatments as types of social experience. Females were reared with their father (paternal exposure) or without their father, and weaning was conducted at 21 days after the birth of the pups. After weaning, juvenile mice lived in sibling groups for 14 more days and then female subjects were either socially housed (two female mice per cage) or individually housed (one mouse per cage). Female subjects belonged to one of four different treatments: 9 females were individually housed at 5 weeks of age without their father present from birth until weaning (individually housed without paternal exposure, IH and–PE), 13 were individually housed at 5 weeks of age, but had their father present until weaning (individually housed with paternal exposure, IH and + PE), 13 were socially housed as adults but did not have their father present until weaning (socially housed without paternal exposure, SH and–PE), and 13 were socially housed as adults and had their father present until weaning (socially housed with paternal exposure, SH and +PE). Henceforth, we use abbreviations to refer to the social treatment groups. The difference in group sizes amongst treatment groups was a result of space constraints in our animal facility. Furthermore, we cannot predict how many pups a female will produce making it difficult to balance the numbers in different groups. Before weaning, mice lived with their litter mates, their mother, and the paternally exposed subjects with their father. Mice from all conditions lived in the same colony room and were exposed to visual, auditory, and olfactory signals from neighboring cages. None of the test subjects had previous co-housing or other direct physical interactions with males prior to the trials.

### Playback testing apparatus

**Fig 1:**
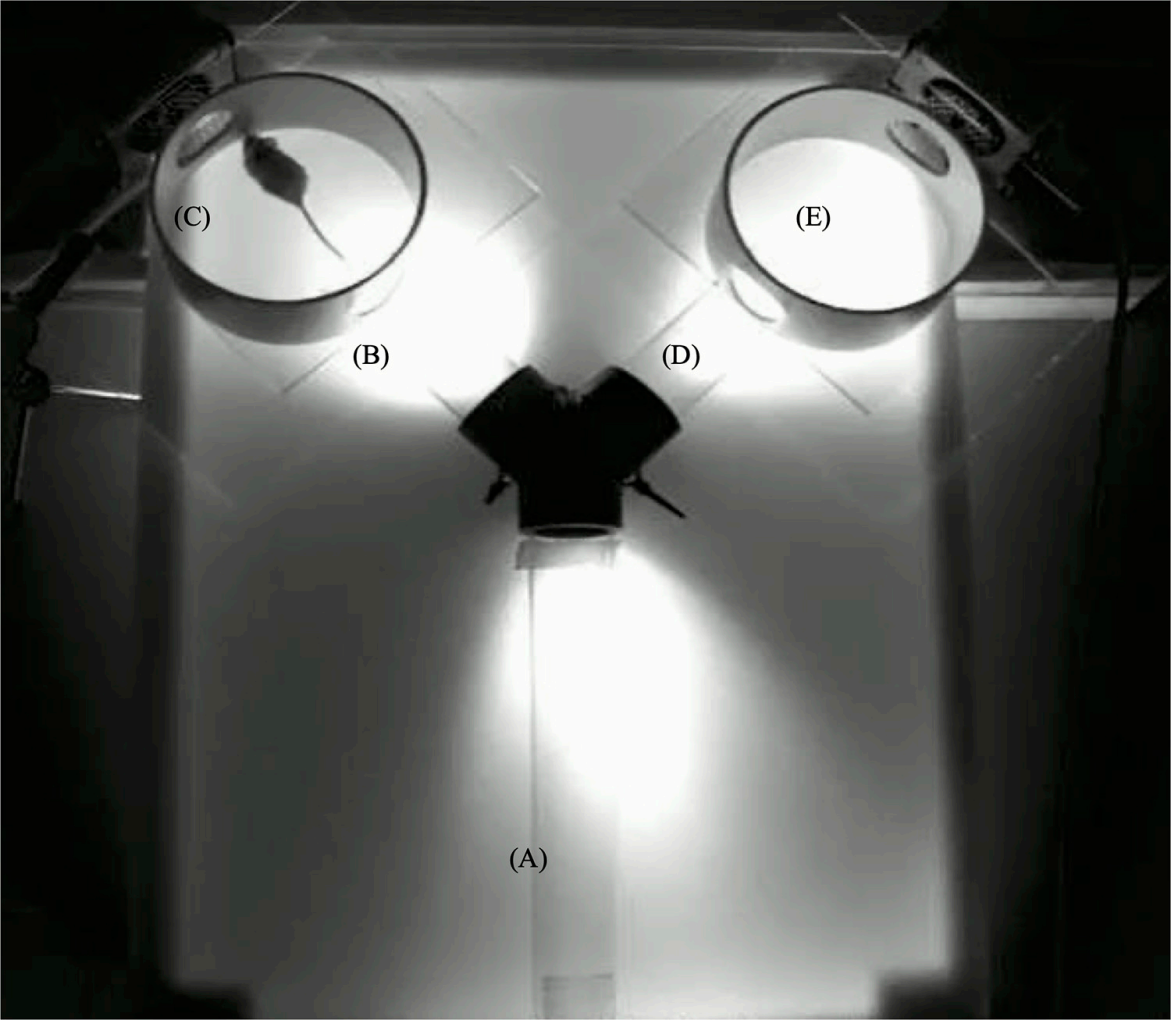
Y-maze apparatus for testing female preferences of playbacks of male USVs versus control. The maze was subdivided into the following sections: (A) a neutral arm, (B and D) two stimulus arms, and each of which is connected to (C and E) a circular arena. Acoustic stimuli, either USVs or control (background noise) were broadcast into the arenas as well as the stimulus arms. For analyses, each stimulus arm and the connected circular arena were combined and defined as one ’stimulus chamber’ (i.e., B and C versus D and E).

Female preference assays were conducted using a Y-maze composed of three transparent plexiglass tubes connected by a non-transparent joint and the apparatus was lit from below with three infrared lights (48 LED Infrared Illuminator) (Fig 1). The Y-maze was modified from a simpler version designed for olfactory experiments [49] by connecting the stimulus arms of the maze to cylindrical arenas (covered with transparent covers on top and bottom), each having a hole for attaching speakers to present acoustic stimuli. Ultrasonic speakers (Ultrasonic Dynamic Speaker Vifa, Avisoft Bioacoustics, Germany) were protected from the mice with wire mesh and were positioned to project playbacks into the arenas and the arms of the maze. Our playback volume settings mirrored the recording sensitivity settings to ensure that our playback amplitudes were within the amplitude range of wild mouse vocalizations. Equal loudness of the playback stimuli was confirmed by emitting the USV and control recordings on both sides of the Y-maze and measuring amplitudes with ultrasound microphones (Knowles FG, Avisoft Bioacoustics, attached to an A/D converter, UltraSoundGate 116H, Avisoft Bioacoustics, Germany) inserted into the maze arms. Playback loudness was adjusted in a way that the microphone would still faintly detect the broadcast stimuli at the junction of the maze, but not in the neutral arm. During each trial, an ultrasound microphone was placed next to the speakers to monitor playbacks from a laptop outside of the experimental room.

### Auditory stimuli: USV playback and controls

In each trial, a subject female was simultaneously presented with two different auditory stimuli: a recording of male USVs on one side of the maze and a control "background noise" on the other side, i.e., the same audio file except with all USV syllables removed. This method ensured that the only differences in the acoustic stimuli were the male USVs. For USV playbacks, we used manipulated audio files created from recordings of male USVs and then played them back in a loop for five minutes into one of the two stimulus chambers of the Y-maze. We used 12 recordings from 12 different primed wild-derived male mice recorded for an unfamiliar and unrelated female behind a divider [20]. In short, vocalizations of the males were recorded on two consecutive days under the following recording regime: On the first day, males were placed into a cage (standard cage type III, Tecniplast, Germany) with an unfamiliar and unrelated female from the same population. The cage was divided by a perforated transparent plexiglass divider. Males were allowed to interact with the females through the divider for 10 minutes. Then, the plexiglass divider was removed, and males and females were allowed to interact directly for 10 minutes, as a method of sexual stimulation (priming) that elicits more vocalizations in the next recording [20]. In the final 5 minutes of recordings, the divider was returned, and males and females were returned to separate sections of the cage. All vocalizations were recorded using an ultrasound microphone (Knowles FG, Avisoft Bioacoustics, Germany). The same recording procedure was repeated for each male on the next day, except a novel female stimulus was presented. The recordings that were utilized for the preparation of the playback files came from the first 10 minutes of recording on day 2. Recordings chosen to be used as playbacks were from the 12 males that emitted the highest number of USVs. We aimed to obtain files with >100 USVs to ensure feasible options for playback file modification. Of the 12 files used, 11 contained at least 116 USVs (range 116–130) but one with only 60 USVs was included to ensure that each of the four different housing treatment groups received the same playbacks. To further standardize playbacks, long pauses lacking male USVs within each recording were cut down to a pause length of maximum 300 ms (previous recordings showed that the mean interbout duration was 300 ms in uncut male vocalizations), ensuring that subjects were presented with consistent auditory stimuli, without long pauses of silence between USVs. The final modified files were approximately 40 s long. Due to variation in the number of females within each treatment group, females from the treatment group “IH and–PE” (n = 9) received 9 unique playbacks, whilst the three other treatment groups (n = 13 per group) received 12 different playbacks with one file being used twice. The creation of all USV playback and control playback files was conducted using the Automatic Mouse Ultrasound Detector (A-MUD) [10] (version 3.2 [20]), which is an extension of the STx signal processing software (Acoustics Research Institute, Vienna). All sounds below 30 kHz were removed from the final playback files using the software SASLab Pro (Avisoft Bioacoustics, Germany), thus excluding the frequencies that typically contain the most intense background noises, as well as audible vocalizations of mice.

### Experimental procedure

We provide a step-by-step protocol summarizing the necessary equipment and explaining how we conduct playback experiments (see S1 Methods in S1 File). Female subjects were sexually primed two days before the experiment with male bedding (15 g of a mixture of bedding from 10 males) and we tested 12 females per day on eight different days. Each female was tested twice on two consecutive days, and all trials were conducted between 15:00 and 18:30, during the dark active period (under red light). To begin each trial, subjects were gently transferred from their home cage to the Y-maze entrance (the neutral arm) using a handling bottle. Once the females voluntarily left the bottle and entered the Y-maze, a trap door was manually shut behind them to prevent returning to the bottle, and the experimenter left the room. The auditory stimuli (both USV and control) were already playing when the females were transferred into the Y-maze. On the second day of female testing, each female received the same stimuli, however, we switched the arms of where the USV and background noise stimuli were emitted. To control for potential side biases of the experimental apparatus, the USV playback was also alternated between arms every four individuals, so that half of the females were first tested with the USV stimulus on either side. Playbacks lasted a total duration of five minutes. Each mouse received its own Y-maze apparatus (clean tubes, lids, joints and arenas) to avoid scent contamination and the trap door was cleaned with ethanol after each trial. The body mass and a vaginal smear was taken from each subject immediately after removal from the Y-maze on trial day 2. As the estrous stages were determined after and not before the experiment (as not to influence female behavior) our groups were not balanced in terms of estrous stage. Vaginal smears were evaluated using a light microscope and each female was assigned to one of four estrous stages [1]. We then classified females into either "sexually receptive" (proestrus and estrus) or "non-receptive" (metestrus and diestrus) estrous stages. These labels refer to the physiological estrous stages when female mice are sexually receptive, and we did not check for behavioral estrus (sexual receptivity *sensu stricto*). We tested the following female groups: IH and–PE (7 receptive, 2 non-receptive), IH and + PE (10 receptive, 3 non-receptive), SH and–PE (9 receptive, 4 non-receptive), SH and + PE (7 receptive, 6 non-receptive). Only data from the second trial day of the experiment were analyzed in the study, as this contained information about the body mass and receptive stage of the female, with the first trial day serving as habituating the females to the testing procedure.

### Recording behavior

The subject females were video recorded during each trial using an infrared sensitive camera (D-Link, model DCS-3710) positioned above the Y-maze to capture all behaviors. Behavioral analyses were conducted using the Observer software (Observer XT 7.0, Noldus, Netherlands). Recordings were blindly analyzed by two experimenters at half speed. We define "chamber” as a stimulus arm and its corresponding circular arena. The duration of time spent in each zone of either side was recorded and summed to generate our response variables defined as “USV chamber” or the control, “background noise chamber”.

### Statistical analyses

All statistical analyses were conducted using R (version 1.2.1335) [50], and we adopted a mixed model approach to incorporate the effects of both fixed and random factors. The choice of either a linear mixed model (LMM) or a generalized linear mixed model (GLMM) was subject to meeting assumptions of normality including homogeneity of residual variance. LMMs were preferentially chosen and GLMM were used only when data could not be transformed to reach normality. For all models, we used a backwards stepwise model selection process to select for the simplest model of best fit. All data were included across the analysis and there were no outliers. Previous studies have shown that females habituate to playbacks and there is still some debate as to how long USV playback studies should last [4,26,28,29]. To address this question, we investigated the results of females’ responses to male USV playbacks over the entire five minutes of each trial and for the first minute. Data for the one-minute analysis met all the assumptions of normality and homogeneity of variance and thus a linear mixed effect model (LMM) with a gaussian distribution was used to conduct a two-way mixed model Analysis of Variance (ANOVA). Data over five-minutes did not meet the assumptions of normality and could not be transformed. Instead, a generalized linear mixed effect model (GLMM) was used with a gamma distribution taking the absolute value of time or SPD to conduct a two-way mixed model Analysis of Variance (ANOVA).

We tested whether females from the different treatment groups spent more time investigating the USV playback chamber or the control chamber and we also tested the intensity of their preference by comparing the ratio of time spent in the two different chambers across treatments (Side Preference Difference or SPD). SPD was measured as the time spent in the control chamber of the Y-maze subtracted from the time spent in the USV chamber:

Sidepreferencedifference(SPD)=TimespentinUSVchamber−Timespentincontrolchamber

Thus, positive values indicate that the female spent more time in the USV chamber of the Y maze, whilst negative values indicate that they spent more time in the control chamber. The further the value is from 0, the stronger the preference. The time spent in the neutral chamber was not included for this section of the analysis as we were solely investigating the difference in female preferences for either of the two different playback stimuli, and not for how long females were uninterested in either stimulus.

To test whether females spent more time in either the USV or background noise chamber, the fixed effects used in the models were chamber (USV playback or control), social experience (SH and + PE, SH and–PE, IH and + PE, IH and–PE) and receptivity (non-receptive or receptive), with mouse family and mouse ID as the random factors, and time as the response variable. We also looked at the effects of housing conditions (SH, IH) and paternal exposure (+ PE,–PE) as their own fixed effects. To compare the intensity of female preference using SPD, a similar approach was used. This time, SPD was the response variable, the same fixed effects were used, except the fixed effect “chamber” and the random effect “mouse ID” were removed from the model as using the response variable SPD renders them redundant.

To determine whether social experience affected whether females habituated to male USV playbacks, females from all different social treatments were analyzed separately. To do this analysis, a response variable named USV preference ratio (UPR) was calculated and defined as the total time spent in the USV chamber over the total time spent in all chambers.

$$USV\;preference\;ratio\;(UPR)=\frac{Time\;spent\;in\;USV\;chamber\;}{Total\;time\;spent\;in\;all\;chambers\;(60s)}$$

Here, we chose to also include the time spent in the neutral chamber as it fully encompasses the entire time when females were not interested in male USV playbacks. For example, a female may spend an increasing amount of time in the neutral chamber (not just the control chamber) if she lost interest in the USV playback, and we still consider this to be evidence of habituation.

All treatment groups met all assumptions of normality, including homogeneity of variance (or were transformed to do so) and thus we used a LMM for each treatment group (SH and + PE, SH and–PE, IH and + PE, IH and–PE) with UPR as the response variable, “minute block” as fixed effect, and mouse ID as random effect to control for the repeated measures design of the analysis. Marginal R^2^ values were reported over conditional R^2^ values using the *r*.*sqauredGLMM* function from the *MuMin (version 1*.*43*.*17)* package, as the purpose of the analysis was to explore the fixed effect of minute block on UPR over any additional random effects. P values were obtained from summary result of the LMM.

Next, we also explored whether housing conditions affected the body mass and estrous cycle of females and whether this would have any implications on female playback preference. Differences in body mass between the various social treatment groups were investigated using a LMM with the response variable body mass, the fixed effects chosen were either overall social background, housing treatment, or paternal exposure and the random effect was age (to control for the variation in body mass not attributed to housing condition). To investigate whether the proportion of receptive or non-receptive females differed within housing treatments, binomial tests were conducted. To determine whether any differences in the proportion of receptive and non-receptive females were due to their housing treatment, we followed up binomial tests with a binomial LMM.

Finally, we explored the effect of estrous stage on SPD separately amongst the SH and IH treatment groups using a LMM and a one-factor ANOVA design. This was done because the proportion of receptive (proestrus and estrus) females to non-receptive (diestrus and metestrus) females across housing treatments was unbalanced (see Fig 6).

## Results

### Females’ overall preferences

**Fig 2:**
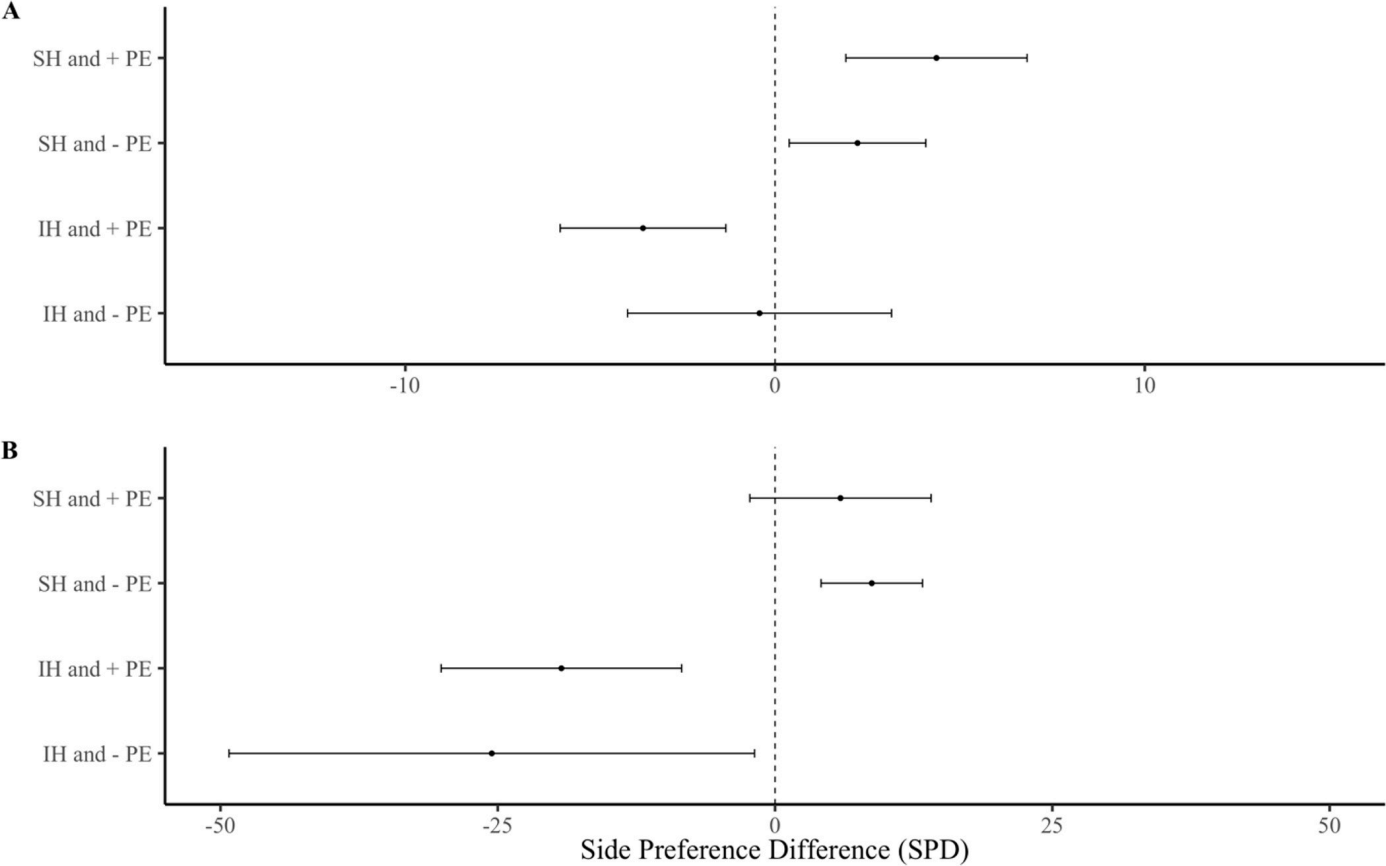
Side preference difference (SPD) of females from different social treatments. Female responses over **(A)** the first min and **(B)** the entire five min playback. Error bars show mean ± SE. Group sizes; SH and + PE (n = 13), SH and–PE (n = 13), IH and + PE (n = 13), IH and–PE (n = 9).

First, we compared the differences in females’ responses to USVs among the four different social treatments (SH and + PE, SH and–PE, IH and + PE, IH and–PE). Our results overall indicated that the 48 females showed no significant preference between the USV playback or the control stimulus over the entire five min (F _(1,43)_ = 0.63, p = 0.432) or the first one min (F _(1,43)_ = 1.32, p = 0.256). There was also no significant interaction between the females’ overall social housing and their preference for either the USV playback chamber or the background noise chamber at five min (F _(3,43)_ = 1.12, p = 0.295) or at one min (F _(3,43)_ = 1.64, p = 0.194). There was a significant difference in SPD from females of different social treatments over five min (Fig 2B; F _(3,44)_ = 2.95, p = 0.043), although Tukey post hoc tests revealed no pairwise contrasts at α <0.05. This result was consistent, although not significant, in the first min (Fig 2A; F _(3,40)_ = 2.21, p = 0.102).

Next, we investigated whether females habituated to male USV playbacks. SH females spent less time in the male USV playback chamber over time; SH and + PE (Fig 5A; R^2^ = 0.04, p = 0.026), SH and–PE (Fig 5B; R^2^ = 0.07, p<0.01), however, this pattern was not observed amongst the IH treatment groups IH and + PE (Fig 5C; R^2^ <0.01, p = 0.442), IH and–PE (Fig 5D; R^2^ = 0.02, p = 0.164).

There were no significant differences in body mass between females from the four different overall social housing treatments (IH and–PE, IH and + PE, SH and–PE, SH and +PE), (F _(3,42)_ = 1.09, p = 0.362).

### Social housing treatments

**Fig 3:**
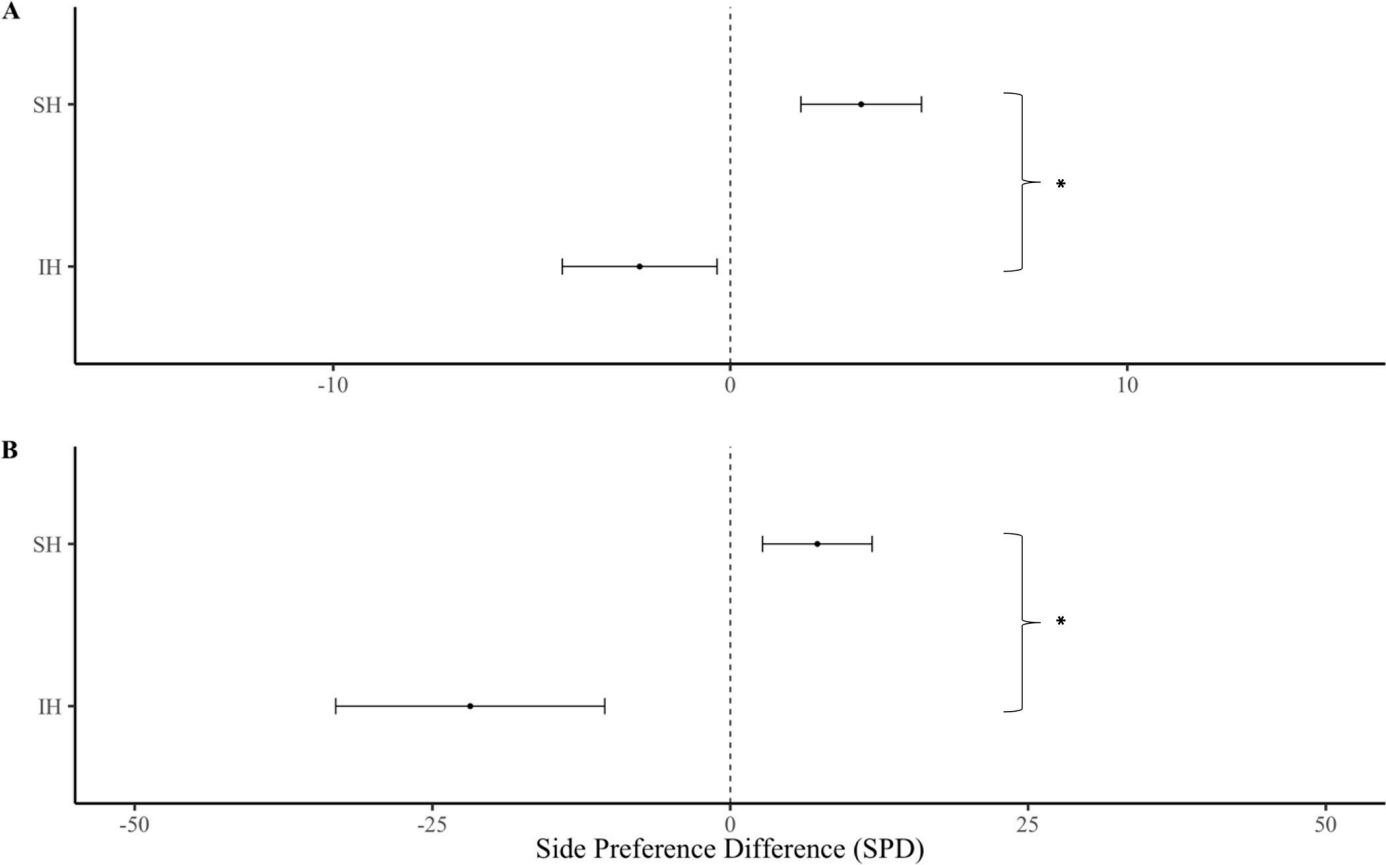
Side preference difference (SPD) of females from different housing treatments regardless of paternal exposure. Female responses over **(A)** the first min and **(B)** the entire five min playback. Error bars show mean ± SE. Asterisks show significant differences. Group sizes; SH (n = 26), IH (n = 22).

There was no significant interaction between housing condition (regardless of paternal exposure) and preference for either the USV playback or control chamber over five min (F _(1,46)_ = 2.86, p = 0.098), although there was a significant interaction over the first min (F _(1,46)_ = 5.25, p = 0.027). SH females spent more time in the USV playback chamber than IH females. SH females had a significantly higher SPD than IH females over both, five min (Fig 3B; F _(1,44)_ = 5.85, p = 0.020) and the first min (Fig 3A; F _(1,43)_ = 6.64, p = 0.015).

There was no statistically significant difference in body mass between IH and SH females (F _(1,45)_ = 3.12, p = 0.084).

### Neonatal paternal exposure

**Fig 4:**
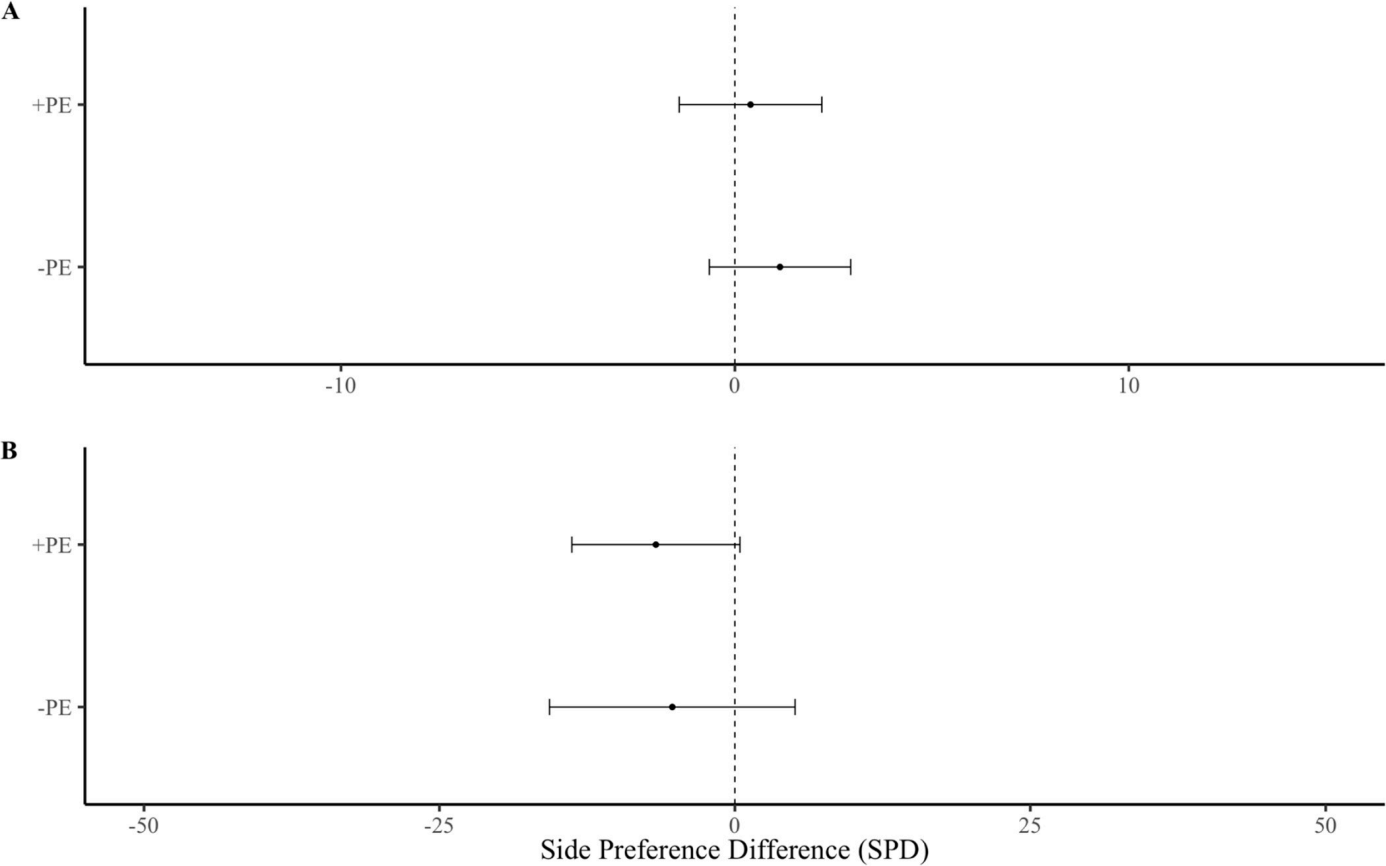
Side preference difference (SPD) of females from different paternal exposures regardless of housing treatment. Female responses over **(A)** the first min and **(B)** the entire five min playback. Error bars show mean ± SE. Asterisks show significant differences. Group sizes: + PE (n = 26),–PE (n = 22).

There was no significant interaction between paternal exposure and female preference for the USV playback stimulus or the control stimulus over five min (F _(1,44)_ = 0.02, p = 0.915), or the first min (F _(1,44)_ = 0.88, p = 0.767). There was no significant difference in SPD between females from different paternal exposures over five (Fig 4B; F _(1,44)_ <0.01, p = 0.980) or one min (Fig 4A; F _(1,43)_ = 0.16, p = 0.691). There was no significant difference in body mass between + PE and–PE females (F _(1,44)_ = 0.07, p = 0.800).

### Sexual receptivity

There was no significant interaction between female sexual receptive stage and their preference for either the USV playback stimulus or the control over five min (F _(1,46)_ = 0.10, p = 0.750); however, there was a significant interaction over the first min (F _(1,46)_ = 4.29, p = 0.044), as non-receptive females spent more time in the USV chamber than receptive females. There was no significant difference in SPD from females in different receptive stages over five min (Fig 6B; F _(1,43)_ = 0.66, p = 0.422) or one min (Fig 6A; F _(1,44)_ = 3.16, p = 0.084).

There was no significant interaction between housing treatment and receptive stage on SPD over five min (F _(1,43)_ = 0.10, p = 0.753) or one min (F _(1,43)_ = 3.32, p = 0.076). Binomial tests revealed a higher proportion of receptive females over non-receptive females within the IH treatment group (Fig 7; p = 0.017), though this was not the case for SH females (Fig 7; p = 0.327). To determine whether this difference in ratio was subject to housing conditions, we followed up this result with a binomial regression, which revealed that these proportions were not significantly different from each another (F _(1,46)_ = 1.35, p = 0.250). When exploring the effects of receptive stage on SPD amongst SH, we found that non-receptive females from the SH treatment group had a significantly higher SPD than receptive females (Fig 8; F _(1,23)_ = 8.4, p<0.01), although this pattern was not observed for IH females (Fig 8; F _(1,18)_ = 0.11, p = 0.746).

## Discussion

Overall, female subjects were not more attracted to playbacks of male USVs than background noise (control), which contrasts with previous studies [4,24], though most studies use silence or white noise as a control and often provide male odor as an enhancing stimulus. Yet, SH females showed preferences for male USVs versus controls, whereas IH females showed the opposite response (Fig 3), which explains why there was no overall effect. We found no evidence that early paternal exposure influenced female attraction to male USVs (Fig 4), contrary to the sexual imprinting hypothesis. Positive imprinting on male USVs has been suggested to facilitate species recognition [27], and negative imprinting has been proposed to mediate inbreeding avoidance [4,28]. However, definitive tests of these hypotheses requires comparing female response to USVs of different males. Estrous stage influenced female attraction for male USVs, as females in non-receptive estrous stages were more attracted to male USV playbacks than those in sexually receptive estrous stages [28] (Fig 6). An interaction between the effects of housing condition and estrous cycle on USV preferences was not detected, potentially due to insufficient sample size or an unbalanced design, and thus, we cannot rule out that these factors might work in combination. Our results indicate that female attraction to male USVs depends upon social housing and estrous stage but does not appear to require paternal exposure. Below we address each of our main findings in more detail.

### Social housing

**Fig 5:**
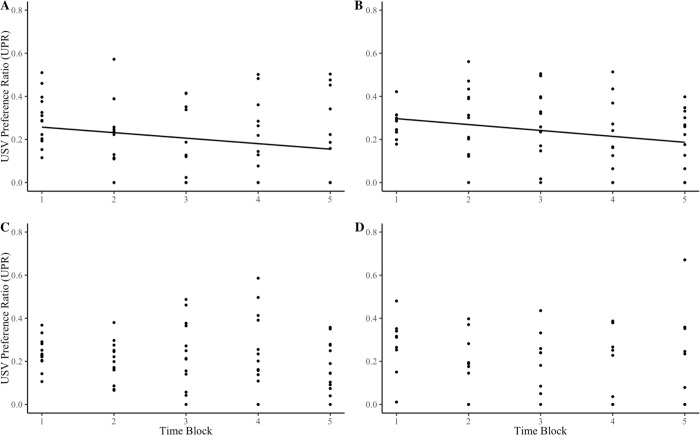
Females’ preferences for male USVs over one min intervals. Results of females socially housed and reared **(A)** with father (n = 13) and **(B)** without father (n = 13); and individually housed and reared **(C)** with father (n = 13) and **(D)** without father (n = 9). Preferences measure using USV preference ratio (UPR) and mean linear regression lines show significant differences. Data points displayed are the UPR of each female at each min block.

Social housing not only influenced female responses in our study, it was necessary for females to show attraction towards playbacks of male USVs. SH females showed preferences for USVs over both the first minute and the entire five minutes of the trial (Fig 3), whereas previous playback studies [4,26,28,29] found that females rapidly habituate to playbacks over time. In contrast, IH females showed a preference for background noise over male USVs, responding as if they perceived male vocalizations as aversive. Females from the SH treatment groups showed habituation and spent less time in the USV chamber over time (Fig 5), this was not the case for the IH treatment group, which is to be expected as they did not initially show a preference for male USVs. It is unclear why females quickly habituate to USV playbacks, though they may lose interest because they quickly realize that no mice are present.

In contrast, a previous study found that social housing had no effect on female attraction to playbacks of USVs [32], and IH females and SH females (housed with females but not females housed with males) showed increased attraction to USVs over time rather than habituation [4,26,28,29]. The difference in our results could be explained by one or more methodological differences, i.e., we studied 48 wild female *Mus musculus musculus* (versus 18 female CBA mice), and we evaluated their estrous stage. We used recordings from 12 males (versus one individual male) and background noise as a control (versus white noise), and we tested female discrimination in a Y-maze (versus a three-chamber assay). Also, the rearing and social housing conditions of the mice differed between these studies.

Previous studies with laboratory mice have found that social housing induces several changes that could explain our results by altering: (1) female sexual receptivity (i.e., IH females are less likely to display sexually receptive lordosis posture during sexual interactions than SH females) [36]; (2) auditory perception of USVs (i.e., female IH mice require more time to learn to discriminate a pure tone, though differences disappeared after a few training sessions) [49]; (3) auditory neurons of the brain (females [38,41], males [51]); and functional neuro-connectivity (i.e., dependencies among remote neurophysiological events [52] was is lower in IH versus SH male mice when exposed to a playback of a female broadband vocalization [40]). Social housing has also been shown to influence hormone levels (i.e., IH females and males have lower corticosterone levels than SH mice [53,54] (but see [55]), and other behaviors, such as hyperlocomotion and increased anxiety/impulsivity in an elevated plus maze test, impaired learning (object recognition) and other aspects of cognition [56], though not always [57]). However, it is premature to generalize these results, as the effects of social housing are often strain-, assay, and sex-specific [58], and can depend upon the timing and duration of the treatments.

The effects of IH on female responses to USVs and other behaviors might be a laboratory artifact due to rearing animals in artificial social conditions. For example, IH results in increasing USV emission due to inducing same-sex mounting during social interactions (male-male mounting in CBA mice [59]; female-female mounting in B6 mice [60]). However, contrary to what is often assumed, not all changes from IH are necessarily pathological nor maladaptive. Female mice in the wild potentially benefit by being cautious when approaching unfamiliar mice when they are solitary, such as during dispersal or in low population densities. Similarly, females can potentially benefit by altering their estrous cycling depending upon the number and sex of conspecifics [61,62] (see below). Little is known about the fitness consequences of changes induced by socio-sexual experience. One study found that keeping male house mice in IH versus SH (with females) had no detectable affect their mating or reproductive success in a female mate choice experiment [63]. Regardless, mice kept in IH versus SH show many unexpected differences in their vocal communication, including USV emission [19,59,60], auditory perception [64], auditory neural pathways [39–41], and responses to USVs, which researchers should consider in future studies.

### Paternal exposure

We found no evidence that neonatal paternal exposure influenced females’ preferences for male USV, suggesting that PE is not necessary for females to develop USV preferences. A previous study found evidence that female preferences for male USVs are acquired during early development, and that paternal exposure prior to weaning increases female responsiveness to unfamiliar USV playbacks [28] (whereas in birds, females positively imprint on their fathers’ vocalizations [63,65,66]). Unlike this previous study, however, we did not compare female preferences for the vocalizations of familiar versus unfamiliar males. Therefore, our results suggest that neonatal exposure to male USVs are unnecessary for females to develop preferences for (unfamiliar) USVs; but they do not rule out sexual imprinting [28]. It is possible that PE had no effect in our study because subjects shared a colony room with other mice and were likely exposed to the USVs of other adult males in neighboring cages. This type of indirect exposure may have been sufficient for females to develop a preference for male vocalizations, and thus, we do not rule out the hypothesis that exposure to USVs influences the development of USV preferences. Prior to weaning, females in our study were also exposed to their litter mates’ USVs, which may be sufficient to induce attraction to male USVs, though pup vocalizations differ from adult males [67].

### Sexual receptivity

**Fig 6:**
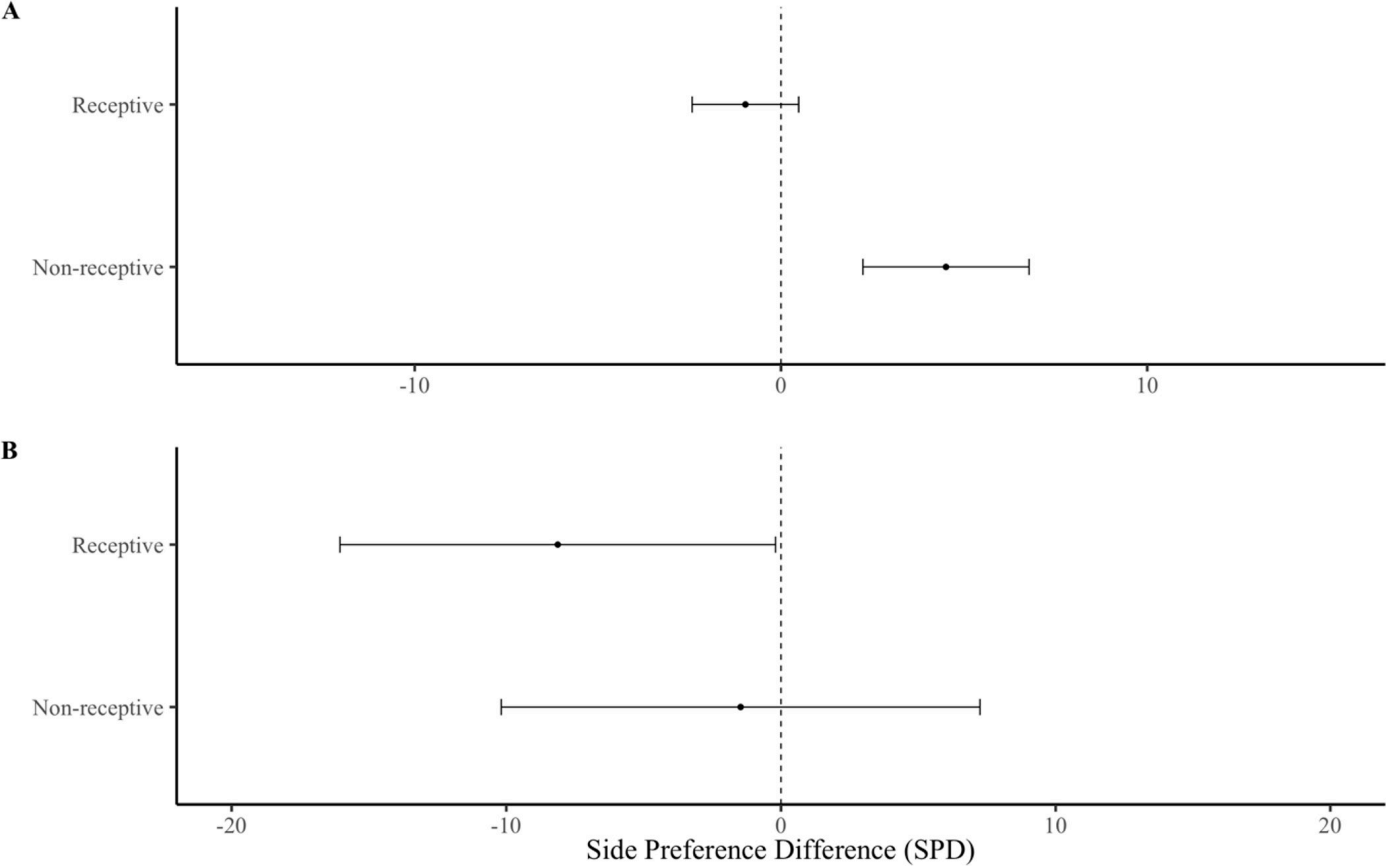
Side preference difference of females in different receptive stages regardless of social treatment. Female responses over **(A)** first min and **(B)** entire five min playback. Error bars show mean ± SE. Asterisks show significant differences. Group sizes: Receptive (n = 33), non-receptive (n = 15).

We found that female attraction to male USVs was affected by their sexual receptivity, but only in the first minute and not over the entirety of the trial. The physiological relevance of a brief attraction to male USVs is unclear, yet we still chose to interpret this result to explore the effects of female sexual receptivity on their preferences for male USVs. It is possible that the effects of female estrous stage for male USVs are most prominent at the beginning of the trial, as this is prior to any possible effect of habituation [4,26,28,29]. We found that non-receptive females (diestrous and metestrous stages) spent a significantly longer time in the USV chamber than receptive females (proestrous and estrous stages) (Fig 6). Our result is consistent with a study on laboratory mice that found females showed preferences for male USVs from a different strain, but only during diestrus; not estrus (though estrous stage was evaluated 5 to 7 h before trials) [28]. Subsequently, these authors confirmed that females show preferences for playbacks of male USVs from a different strain during diestrus [68]. Females might be most attracted to male USVs before estrus because they begin assessing potential mates during this period (wild house mice in seminatural conditions usually take several weeks to become pregnant [49,69]). However, this interpretation does not explain why females were not attracted to male USVs during estrus. It has been shown that social investigation towards unfamiliar males is enhanced by moderate levels of estrogen [70] and sexually receptive (estrus) females in other species show stronger preferences for male odor than non-receptive females [71–73], but why female responses for male USVs are reversed is unclear. Asaba et al [28] proposed that females have two systems for mate preferences: the first is a preference for masculine males (social rank or higher testosterone) in the estrous phase [73–75]; the second is inbreeding avoidance in the diestrous stage [28,68]. A previous study found that females in estrus were preferentially attracted to the USVs of unfamiliar non-kin over those of familiar kin [4], though they were not compared with non-estrous females and this result is based on a smaller sample size (n = 10) to the previously mentioned study [28] (n = 40). Whilst in our study, the number of vocalizations (which may be influenced by testosterone [76]) was standardized, all recordings came from unrelated males. Thus, to test the inbreeding avoidance hypothesis, future studies comparing female USV preference to familiar vs unfamiliar kin amongst females of different estrous stages is required. Moreover, studies are needed to manipulate estrous stage experimentally to test its effects on female responses to male USVs.

### Social housing and sexual receptivity

**Fig 7:**
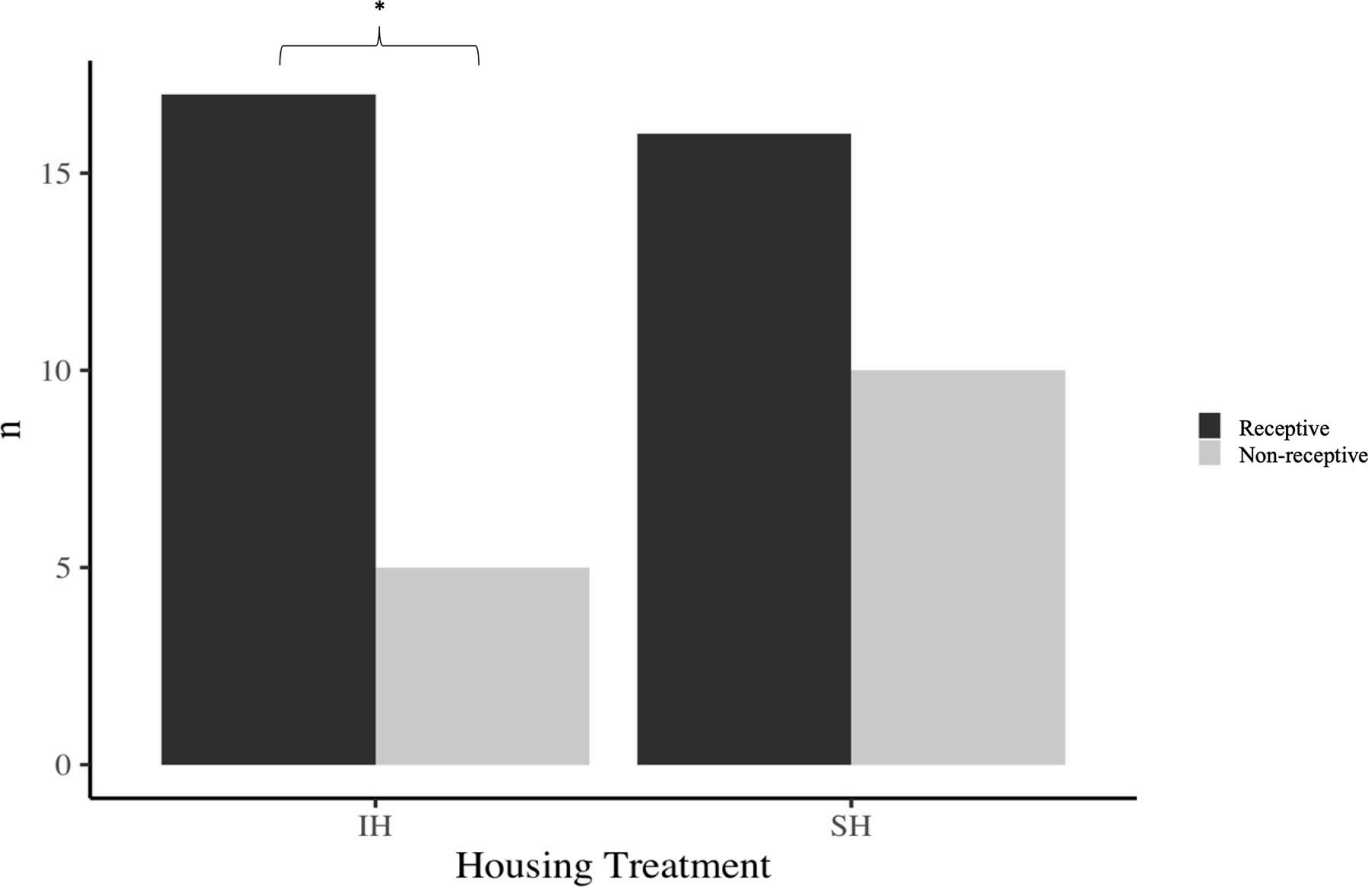
Number of females in sexually receptive and non-receptive stages comparing individually housed (IH) versus socially housed (SH) conditions. Asterisks show significant differences. Group sizes; SH and receptive (n = 16), SH and non-receptive (n = 10), IH and receptive (n = 17), IH and non-receptive (n = 5).

**Fig 8:**
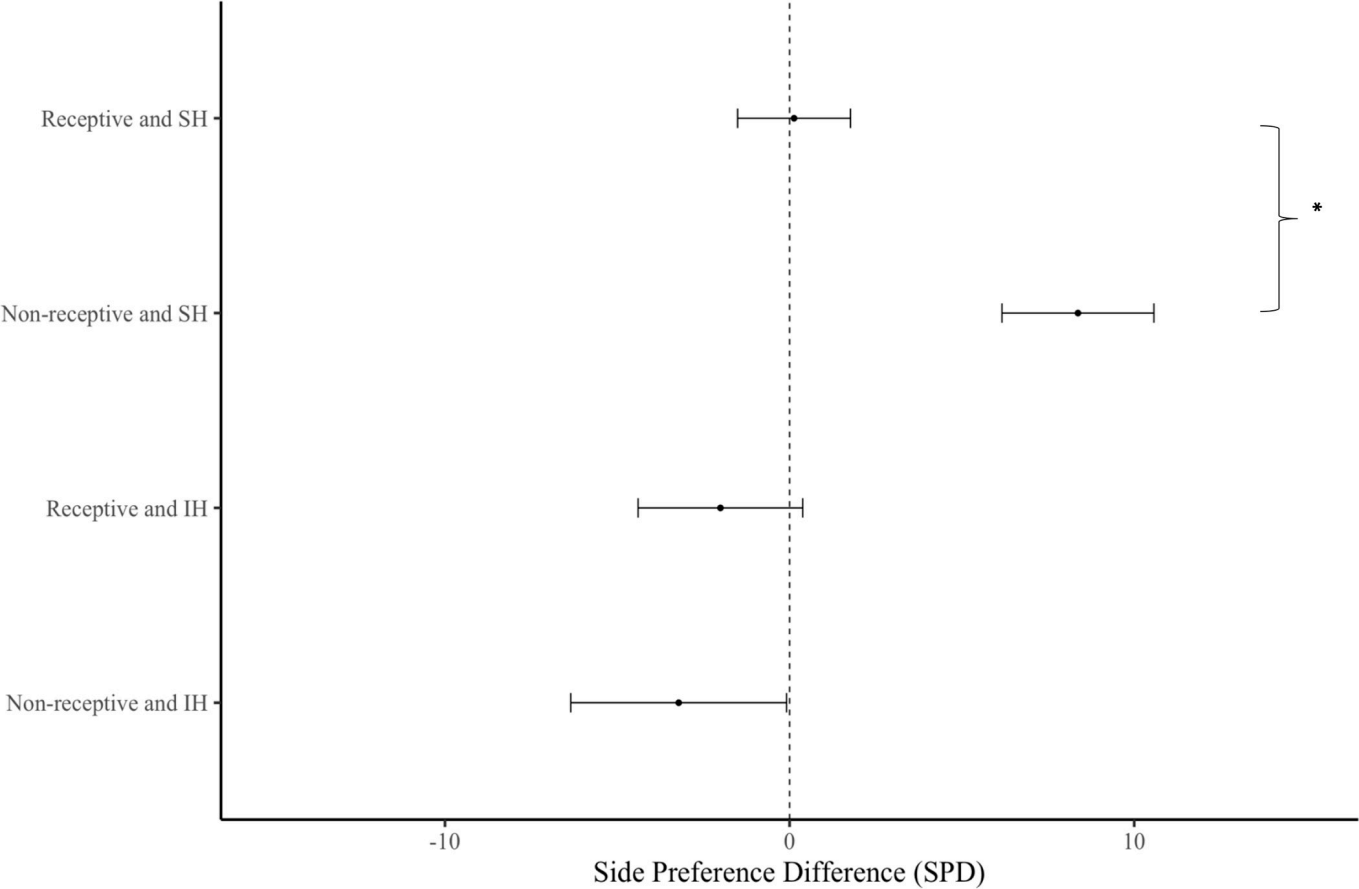
Side preference difference of females in different receptive stages across different housing conditions. Error bars show mean ± SE. Asterisks show significant differences. Group sizes; SH and receptive (n = 16), SH and non-receptive (n = 10), IH and receptive (n = 17), IH and non-receptive (n = 5).

In addition to testing the effects of housing, paternal exposure, and estrous cycle on female proclivity to investigate male USVs, we also investigated whether these factors interact. We found that a higher proportion of IH females were in receptive estrous stages than non-receptive estrous stages, although this was not the case for SH females (Fig 7). Amongst SH females, those in non-receptive estrous stages showed greater USV preferences than those in receptive stages, but there was no effect of sexual receptivity on USV preference amongst IH females (Fig 8). There was no significant interaction between housing type and estrous stage on female USV preference when investigating the entire sample population.

The influence of housing conditions on estrous cycling has been well documented in house mice, as females housed in groups and isolated from males show suppression or prolongation of estrous cycles (Lee-Boot effect) [61]. Isolated females tend to display short estrous cycles of 4–6 days [77], whereas pair-housed females are lengthened slightly, and group-housed females (4–6 individuals) have even longer cycles [78] with complete suppression occurring once all-female groups become even larger [79]. To our knowledge, no study has previously examined whether the Lee-Boot effect influences female preference for male USVs. Exposure to male scent induces estrous cycling in wild house mice [62], and we exposed females to male bedding 48 h before trials to ensure cycling. Therefore, our result suggests that the estrous cycles of IH females were shorter than those of SH females, consistent with the Lee-Boot effect. However, further investigation of the regression analysis suggests that these perceived differences in estrus ratio are not due to housing treatment and so we are unable to make strong conclusions.

Although the interaction between social housing and estrous cycle was not statistically significant (p = 0.076), this result may be a consequence of an imbalanced design and using an equal distribution of non-receptive and receptive females might provide a positive result. This is because the effect of estrous cycle on female USV preference was significant when SH housed females were analyzed independently, which consisted of a more balanced design. Thus, our results suggest that housing condition and estrous stage work in combination, rather than independently to affect female USV preferences, at least amongst SH females (this was not the case for IH females, however). It is difficult to disentangle the independent effects of housing and estrous stage, as the Lee-Boot effect has been shown in wild house mice [60], as well as laboratory mice [61,77–79]. Many of the differences in socio-sexual behaviors found between SH and IH females in other studies [36,38,64] did not report the estrous stage of the mice [36,64] or an unbalanced design was used [38]. In our study, we used 26 SH (16 receptive, 10 non-receptive) and 22 IH females (17 receptive, 5 receptive), and thus studies using a larger sample size and a balanced design–as well as experimentally manipulating estrous stage–are needed to investigate this potential interaction.

## Conclusions and future directions

Our results provide the first evidence to our knowledge that female house mice are more likely to show attraction towards male USVs if they are socially housed, whereas individually housed females are more likely to avoid them. We found no effect of neonatal paternal exposure on female preference for male USV playbacks, though this result does not rule out sexual imprinting (and may be due to females in our study being exposed to auditory cues of adult males in our colony room). We found that non-receptive females show greater preferences for male playbacks than sexually receptive females, and this result was most pronounced amongst SH females and did not appear amongst IH females. Future studies are needed to investigate the effect of behavioral estrus (sexual receptivity *sensu stricto)* on female attraction to male USVs, and using a within-subject design.

Our findings contribute to a growing number of studies showing that social housing [33] and estrous stage [28,46] influence the behavior of house mice and raise the possibility that these factors may interact. Studies are now needed to explore the ecological relevance of these results, such as by varying the number individuals, density, and sex ratio in natural or seminatural conditions [69], and not merely comparing the effects of IH versus SH in small cages [45]. Future research is also needed to investigate the effects of the timing and duration of social housing and estrous stage on behavior (i.e., ontogeny), and underlying proximate mechanisms, including endocrine responses [53], sensory perception [37], neural pathways [39–41] and their adaptive consequences, such as altering sexual motivation [36] and mating success and reproductive success [63].

Although results from studies on social housing have prompted recommendations to stop the use of IH in animal research [80], such prescriptions are too general and overly simplistic [81], especially for wild house mice, which are highly territorial and aggressive towards male cage mates. Furthermore, the various effects of IH are not necessarily pathological, contrary to what is generally assumed (e.g., solitary females may benefit by being cautious about approaching the USVs of unfamiliar mice). To better understand why many behaviors depend upon social housing and experience, more studies are needed to determine the fitness consequences of altering behavior in response to social experiences and social information (e.g., social competence) [82].

Finally, publications of studies on rodents should provide more specifics about housing conditions and estrous status, as our results show that these sources of variation can influence behavior. It is important to note that if social housing had not been recorded in our study, we would have erroneously concluded that females show no attraction to male USVs, i.e., a false negative. Thus, improving the reporting of variables such as these that have been shown to influence behavior and physiology should help to address the replication crisis [42–45].

## Supporting information

S1 File Protocol for conducting playback experiments. (PDF)

S2 File Data file: USV preferences raw data- one min analysis. (TXT)

S3 File Data file: USV preferences raw data- five min analysis. (TXT)

S4 File Data file: USV preferences raw data- habituation. (TXT)
