# Supplementary material for: Attraction of female house mice to male ultrasonic courtship vocalizations depends on their social experience and estrous stage
Source: PLoS One. 2023 Oct 10;18(10):e0285642. doi: 10.1371/journal.pone.0285642 (PMC10564145; doi:10.1371/journal.pone.0285642)
Supplement: S1 File — (PDF) [file pone.0285642.s001.pdf]

Supplemental information

Attraction of female mice to male ultrasonic courtship vocalizations depends on their social experience and estrous stage

Jakob Beck, Bettina Wernisch, Teresa Klaus, Dustin J. Penn*, Sarah M. Zala

Konrad Lorenz Institute of Ethology, Department of Interdisciplinary Life Sciences, University of Veterinary Medicine Vienna, Vienna, Austria

* Corresponding author: Dustin Penn (dustin.penn@vetmeduni.ac.at)

S1 Methods

Protocol for conducting playback experiments

Here we provide a protocol for conducting playback experiments to summarize necessary equipment and procedures used. Information about other procedures, such as recording and processing USVs male mice for playbacks, priming females, and estrous staging are provided in the Methods section. Trials should be conducted in an experimental room (ER) with no observers present, and the mice should be observed from a separate room with a video camera (so that mice cannot hear, see or smell observers). The procedures used here are similar to previous playback studies conducted on wild house mice in our laboratory [1, 2] and to another protocol developed for laboratory mice [3], though these previous studies used a different testing apparatus and included olfactory stimuli.

A. Equipment checklist

- 1 or more Y-mazes (see Figure 1). We use a different maze for each trial because otherwise the apparatus must be cleaned in between each trial.
- 2 Ultrasonic speakers (Ultrasonic Dynamic Speakers Vifa, Avisoft Bioacoustics, Germany)
- 2 UltraSoundGate Players 116H (Avisoft Bioacoustics, Germany)
- 1 Ultrasound microphone (Knowles FG, Avisoft Bioacoustics, Germany) held by desk tripod.
- 1 Infrared-sensitive video camera (D-Link, model DCS-3710).
- 2 Laptop computers with playback software Avisoft Recorder USGH (Avisoft Bioacoustics, Germany) and files for playbacks (modified recordings of males), and for analyzing behavior (Observer XT 7.0, Noldus, Netherlands)
- Handling Bottles (HBs) for transferring mice from cage to Y-maze
- Equipment for vaginal smears: handling bag for mice, light microscope, distilled water, loops, and slides.
- Changing Box (used for wild mice which otherwise often escape after opening cage)
- Experimental data sheet
- Stop watch
- Cleaning materials: 70% EtOH and paper towels
- Safety equipment: Lab coat and gloves

B. Preparation before trials:

- Prime female subjects in advance

- Prepare and print experimental data sheet
- Put on lab coat and gloves.
- Prepare clean HBs and clean Y-maze tubes and arenas.
- Start laptops and check all equipment and settings: Loudness and position of the speakers
- Turn on infrared lights of Y-maze.
- Clean remote-controlled door of Y-maze with alcohol.
- Turn off all white lights.

C. Instructions for conducting each trial:

1. Starting a trial

- Turn on red light in Experimental Room (ER).
- Place a clean set of Y-maze tubes onto the recording platform using the tape markings on the platform to position the tubes correctly.
- Check and adjust position of the remote-controlled door at the entry zone of the Y-maze.
- Load the playback file (double check ID and side) into the program (noted on experimental sheet) and start the playback.
- Fetch mouse cage with female subject for the next trial from Mouse Colony Room and position the cage in the Changing Box (used for wild mice).
- Start the video recording.
- Turn off red ceiling light in the ER so that only the infrared lights under the Y-maze are on.
- Double-check female and her ID and gently place HB into her cage and wait for her to enter.
- Once the female enters the HB, cover the opening with your hand, transport female to the ER and place HB in front of the entry tube of the Y-maze.
- Start timer on your stop watch.
- Wait until female enters the Y-maze (If she does not enter within 10 min, gently move the HB to encourage her to exit).
- Once the female is inside the maze, close trap door behind her, leave ER and close the door behind you, reset and start stop watch again. Note times.

2. Running a trial

- Observe video and record notes (on experimental data sheet) and especially any irregularities in the female's behavior.
- Fill out experimental sheet during trial and note the start time of video, record notes about subject's behavior and female's body mass
- After 5 min on stop watch, terminate video recording.

3. After completing a trial

- Enter the ER, turn on red ceiling light, and use HB to remove female from maze.
- On habituation days (when acclimating mice to maze): return female to home cage and colony. On experimental days: tare a HB on the scale, place female into HB, note her body mass, take estrus smear, and return female to home cage and colony.
- Remove Y-maze components and rinse/soak until final wash at the end of the day.
- Clean trap door of Y-maze with alcohol.

D. After all trials completed:

- Wash all of the equipment: Y-maze components, cages, and HBs.
- Make sure all your females have been returned to the colony properly and the water bottles in cages are in place.
- Transfer the recorded videos from the iSpy Folder into your experimental folder on the laptop and also make a backup on external hard drive.

References

[1] Musolf K, Hoffmann F, Penn DJ. Ultrasonic courtship vocalizations in wild house mice, Mus musculus musculus. Anim Behav. 2010;79(3): 757-764.

[2] Musolf K, Meindl S, Larsen AL, Kalcounis-Rueppell MC, Penn DJ. Ultrasonic vocalizations of male mice differ among species and females show assortative preferences for male calls. PLoS one. 2015;10(8): 0134123.

[3] Asaba A, Kato M, Koshida N, Kikusui T. Determining ultrasonic vocalization preferences in mice using a two-choice playback test. J Vis Exp. 2015;(103): 53074.
